# The prevalence and persistence of maternal morbidities after first vs. second birth: A prospective cohort study in Ireland

**DOI:** 10.1371/journal.pone.0332891

**Published:** 2025-10-22

**Authors:** Francesca Wuytack, Brenda Lynch, Patrick Moran, Anthony P. Fitzgerald, Paul Corcoran, Cecily Begley, Deirdre Daly

**Affiliations:** 1 School of Nursing & Midwifery, Trinity College Dublin, Dublin, Ireland; 2 Trinity Centre for Maternity Care Research (TCMCR), Trinity College Dublin, Dublin, Ireland; 3 Department of Management and Enterprise, Munster Technological University, Bishopstown Campus, Cork, Ireland; 4 School of Public Health, University College Cork, Cork, Ireland; 5 Department of Statistics, University College Cork, Cork, Ireland; 6 National Perinatal Epidemiology Centre, Department of Obstetrics and Gynaecology, University College Cork, Cork, Ireland; Kasr Alainy Medical School, Cairo University, EGYPT

## Abstract

**Background:**

This study aimed to assess the prevalence and persistence of key maternal morbidities – urinary incontinence, faecal incontinence, pelvic girdle pain, sexual health problems, depression, and anxiety – after the births of a first and second baby. Its longitudinal design distinguishes it from previous research by examining a range of morbidities over two childbirths and stratifying results based on women’s prior health history.

**Methods and findings:**

A prospective cohort of 3,047 nulliparous women completed surveys in early pregnancy and at 3, 6, 9, and 12-months postpartum after their first birth. Of these, 254 women who had a second baby and consented to follow-up completed additional surveys at 6-months and/or 12-months postpartum after their second baby’s birth. Prevalence of each morbidity was reported at each time point, 3, 6, 9, 12-months after the first birth; and 6 and/or 12-months after the second birth. Persistence was defined as reporting the morbidity at 6 and/or 12-months after the first birth and again at 6 and/or 12-months after the second birth. Among 91 women reporting urinary incontinence after their first baby’s birth, persistence was 100% (n = 5/5) for those who experienced it in the 12-months prior to their first pregnancy and 39.5% (n = 34/86) for those without (RR 2.53, 95% CI (1.95–3.29)). For pelvic girdle pain (n = 86), persistence was 98.1% (n = 52/53) who experienced it in the 12-months prior to their first pregnancy and 97.0% (n = 32/33) for those without (RR 1.01, 95% CI (0.94–1.09)). Sexual health problems persisted in 100% (n = 76/76) of those who experienced it in the 12-months prior to their first pregnancy versus 89.6% (n = 43/48) without (RR 1.12, 95% CI (1.02–1.23)). Depression persisted in 50% (n = 4/8) of those who experienced it in the 12 months prior to their first pregnancy versus 19.0% (n = 15/79) without (RR 2.63, 95% CI (1.15–6.03)); and anxiety persisted in 100% (n = 1/1) of those who experienced it in the 12-months prior to their first pregnancy versus 13.5% (n = 12/89) without anxiety (RR 7.42, 95% CI (4.38–12.55)).

**Conclusions:**

These findings underscore the need for early identification and intervention to mitigate long-term health issues, highlighting the importance of targeted pregnancy and postpartum care for women with prior maternal morbidities.

## 1. Introduction

During pregnancy, giving birth, and in the postpartum period women undergo significant physical, mental, and social transformations. Whilst many women experience excellent health throughout, others may experience short and long-term health problems. Maternity care tends to focus on pregnancy, the birth and the initial six weeks after the birth [1], yet there is growing evidence that maternal health should be considered within the context of the women’s health over their life-course [2]. While the 2019 global burden of disease report found that maternal disorders represent 1.07% (0.94% to 1.21%) of the total DALYs (Disability Adjusted Life Years) [3], pregnancy and birth factors are also associated with a range of other common health conditions throughout women’s lives. Common morbidities include incontinence, pelvic girdle pain, sexual health problems, depression and anxiety. A large portion of the existing literature has focused on maternal mortality [4], and data on maternal morbidity remains sporadic and incomplete [5,6]. This study provides important evidence on six maternal morbidities women experience before and during pregnancy, and after having their first and second baby: urinary and faecal incontinence (UI, FI), pelvic girdle pain (PGP), sexual health problems (SHP), depression and anxiety.

Urinary incontinence (UI) is defined as any involuntary leakage of urine, and the three main types are stress urinary incontinence (SUI) (provoked by physical exertion or effort, including coughing and sneezing); urge urinary incontinence (UUI) (an urgent need to urinate) or a combination of both, mixed urinary incontinence (MUI) [7]. While the prevalence of UI varies depending on the definitions and measurement tools used, overall prevalence is high before and during a woman’s first pregnancy [8] and postpartum. A recent systematic review reported a UI prevalence during pregnancy of 41% (7–75%) based on 44 studies [9]. A longitudinal cohort study conducted with 1,507 nulliparous women in Australia reported a prevalence of UI, defined as leaking urine monthly or more frequently, of 10.8% pre-pregnancy and 55.9% during the third trimester [10,11]. The prevalence of UI ranged from 46.9% to 40.2% between 4 and 12-months postpartum [12]. It is also known that the prevalence of UI increases with age [10,12].

Faecal incontinence (FI) is involuntary leakage of stools and is less common than UI. Women who experience third- and fourth-degree perineal tears, who have an instrument-assisted vaginal birth, or are of older age are more at risk [13]. A systematic review of nine prospective cohort studies [14] identified the following risk factors: FI during pregnancy (Pooled OR 8.90 95% CI (1.62–48.85), 3 studies, 2,582 participants), maternal age over 35 years (Pooled OR 2.16 95% CI (1.57–2.97), 5 studies, 10,986 participants), antenatal body mass index (BMI) >30 kg/m^2^ (Pooled OR 1.78 95% CI (1.09–2.92), 3 studies, 2,829 participants), spontaneous (Pooled OR 1.41 95% CI (1.02–1.96), 4 studies, 10,708 participants) and instrument-assisted vaginal birth (Pooled OR 1.94 95% CI (1.53–2.45), 6 studies, 14,873 participants), labour augmentation with synthetic oxytocin (Pooled OR 2.41 95% CI (1.24–4.67), 2 studies, 4,165 participants), and infant birthweight greater than 4000g (Pooled OR 3.12 95% CI (1.46–6.71), 2 studies, 4,980 participants). This review includes studies describing morbidities associated with two or more pregnancies and/or obstetric-related risk factors for FI (including flatus incontinence). However, multivariable meta-analysis was not possible because of the heterogeneity of the populations and outcome measures, therefore these results were based on univariate meta-analysis, calculating the pool odds ratios.

Pelvic Girdle Pain (PGP) is defined as pain between the posterior iliac crest and the inferior gluteal fold or at the pubic symphysis. Prevalence of PGP during pregnancy varies from 23% to 65% depending on the definition used, and 8.5% of women have significant symptoms 2 years postpartum [15–17]. PGP negatively impacts women’s quality of life during pregnancy [18] and postpartum [19,20].

Pregnancy and birth also impact women’s sexual health with nearly half of women reporting sexual health problems 6 - months postpartum. Common postpartum sexual health problems (SHP) include vaginal dryness, dyspareunia, and a lack of interest in sex [21,22]. Various factors were reported as being associated with postpartum sexual dysfunction and/or dyspareunia in a 2022 systematic review [23]. For example, anal sphincter injury was associated with sexual dysfunction and dyspareunia; episiotomy was associated with dyspareunia but not sexual dysfunction; compared with spontaneous vaginal birth, caesarean section reduced the odds of reporting dyspareunia whilst an instrument-assisted birth increased theodds of sexual dysfunction but not dyspareunia. The authors concluded that perineal trauma rather than mode of birth increased the odds of sexual health dysfunction in the first postpartum year.

Women’s mental health is also influenced by the changes occurring during the perinatal and postpartum period. Depression and anxiety are prevalent peripartum disorders affecting as many as 30% of women [23–25]. Shorey et al. (2022) conducted a systematic review on postpartum depression among healthy women, defining it as women without prior history of mental illnesses (including postpartum depression) and with healthy infants. The incidence of depressive symptoms was 12% (95% CI 0.04–0.20)(58 articles, 37,294 women) while the overall prevalence was 17% (95% CI 0.15–0.20) [26]. A recently published systematic review and narrative analysis on postpartum anxiety (PPA) found that anxiety symptoms before and during pregnancy, and in the early postpartum period, predicted later PPA (16 studies, 13,358 women). Other risk factors included social support and relationship satisfaction, including perceived relationship quality [27]. In Ireland, in the 12-months after their first baby, 9.5% of women reported symptoms of moderate/severe anxiety, 14.2% reported symptoms of depression, and 19.2% reported symptoms of stress, measured on the 21-item Depression, Anxiety and Stress Scale (DASS) [28].

More recently, there is growing recognition of the co-existence of postpartum mental and physical morbidities. For example, women who reported higher depression or anxiety symptom scores also tended to report more severe UI, PGP, and SPH [29]. This bi-directional relationship underscores the need to study mental health and physical health symptoms together, rather than in isolation, to fully understand the trajectories of women’s postpartum health over time.

Previous studies explored the impact of parity on aspects of women’s health and several focused on single health problems. In many studies, increasing parity has been associated with worse or more severe symptoms, but the association is not consistent for all health problems. For example, a recent systematic review found no difference in UI prevalence in primiparous versus multiparous women up to 1 year postpartum [9], whilst an older study found that greater parity was associated with persistent UI up to 12 years [30]. The risk of developing pelvic girdle pain syndrome (pain at both sacroiliac joints and pubic symphysis) increased with parity [31] and women who experienced PGP in previous pregnancies also seem to be more likely to experience PGP in a subsequent pregnancy [31]. In one longitudinal study in France and Italy, primiparous women reported more sexual difficulties postpartum than multiparous women [32].

In relation to mental health, one recent study found that primiparous women had higher anxiety symptoms postpartum than multiparous women but found no difference in depressive symptoms [33]. Women who experienced their first depressive episode during the postpartum period were at an increased risk of postpartum depressive episodes in subsequent pregnancies [34]. There is also evidence that differences exist between primiparous women and multiparous women; while prevalence of postpartum depression symptoms may be higher among multiparous women, especially during the first 6-months postpartum [34], other studies found that differences between women who had their first or second baby were scarce and varied [32].

The consequences of these persistent conditions can impact women’s quality of life and well-being significantly. Morbidities like UI and FI can lead to social isolation [35], SHP may affect relationships [36], and mental health disorders can compound the burden of physical health issues [37]. Addressing these conditions with comprehensive care that extends beyond the immediate postpartum period is essential for improving maternal outcomes.

All of this literature acknowledges that maternal morbidity impacts women’s long-term physical and mental health significantly. However, most studies concentrated either on a single morbidity or examined outcomes only after a first birth, and did not explore how these morbidities persist or evolve through subsequent pregnancies. This gap limits our understanding of the cumulative health burden women may face across multiple pregnancies and postpartum periods. Furthermore, the predictive role of health conditions that pre-exist a woman’s first pregnancy and their association with persistent postpartum morbidities remains under-explored. This study addresses these gaps by prospectively investigating the prevalence and persistence of six common maternal morbidities (UI, FI, PGP, SHP, depression, and anxiety) following both first and second births, stratified according to women’s health in the 12-months before their first pregnancy. By explicitly examining morbidity trajectories over consecutive pregnancies, this research aims to provide crucial insights to identify targeted care strategies for women during pregnancy and beyond.

The objectives of this study were: (1) to determine the prevalence of six maternal morbidities (UI, FI, PGP, SHP, anxiety, depression) before and during pregnancy with the first birth, at 3, 6, 9 and 12-months after the birth, and at 6 and 12-months after the birth of their second baby; stratified by whether or not women reported the morbidity in the 12 months before their first pregnancy, and (2) to determine the prevalence of persistent symptoms for all of these six morbidities

## 2. Methods

### 2.1 Ethical issues

Ethical approval for the MAMMI study was granted by the following research ethics committees (RECs): the Rotunda Hospital on 03/22/2011 [no reference number]; Galway University Hospital on 31/05/2013 [Ref: C.A.900], and the Coombe Women and Infants University Hospital on 02/04/2014 [Ref: Study No. 9–2014] and Trinity College Dublin on 16/05/2011 [No reference number], on 05/03/2014 [Ref: 140905] and on 04/03/2015 [Ref: 141211]. Ethical approval for the MAMMI-SIM study was granted by Trinity College Dublin on 28/07/2017 [Ref: 170603]. Written consent was gained from all women for the MAMMI study and again for the MAMMI-SIM study.

### 2.2 Study design & data collection

We conducted a prospective cohort study, the MAMMI study. The primary goal was to recruit nulliparous women from three of Ireland’s 19 urban-based maternity hospitals, two large (~8,000 births annually) and one medium-sized (~3,000 births annually). All 19 maternity hospitals are ‘urban-based’, some are in cities and others are in large towns. However, they all cater for women with various medical and obstetric risk factors and women can choose to self-refer to any maternity hospital. Recruitment took place between 2012 and 2017 across three sites (Site 1: Jan 2012–Oct 2014; Site 2: Oct 2013–July 2015; Site 3: Aug 2015–Mar 2017). Eligible participants were nulliparous women aged ≥18, able to read and understand English, and booked for care in these hospitals, regardless of medical or obstetric risk factors. Women who experienced miscarriage, stillbirth, or neonatal loss, or whose babies were in Neonatal Intensive Care Unit (NICU) at postpartum follow-up, were excluded.

During the recruitment time period, there were no substantial changes to the organisation of the maternity services in Ireland, or to birth outcomes for women.

There were 70,709 maternities in 2012 and 64,097 in 2016. In 2012, 40.5% of primiparous women with a singleton pregnancy birthed spontaneously, 28.8% had an instrument-assisted birth and 30.3% birthed by caesarean section. In 2016, 36.6% of primiparous women with singleton pregnancy birthed spontaneously, 28.9% had an instrument-assisted birth and 33.9% birthed by caesarean section. The length of stay postpartum was 0–2 days for 80.6% women in 2012 and 78.8% of women in 2016. [38,39]

The midwives gave women the study information pack at their booking visit and asked them if they were willing to be contacted by a researcher, to answer questions and find out if they might be willing to take part. Over the duration of the recruitment period, approximately 8,240 women were offered the study information and 3,047 women were enrolled in the MAMMI study.

**Fig 1:**
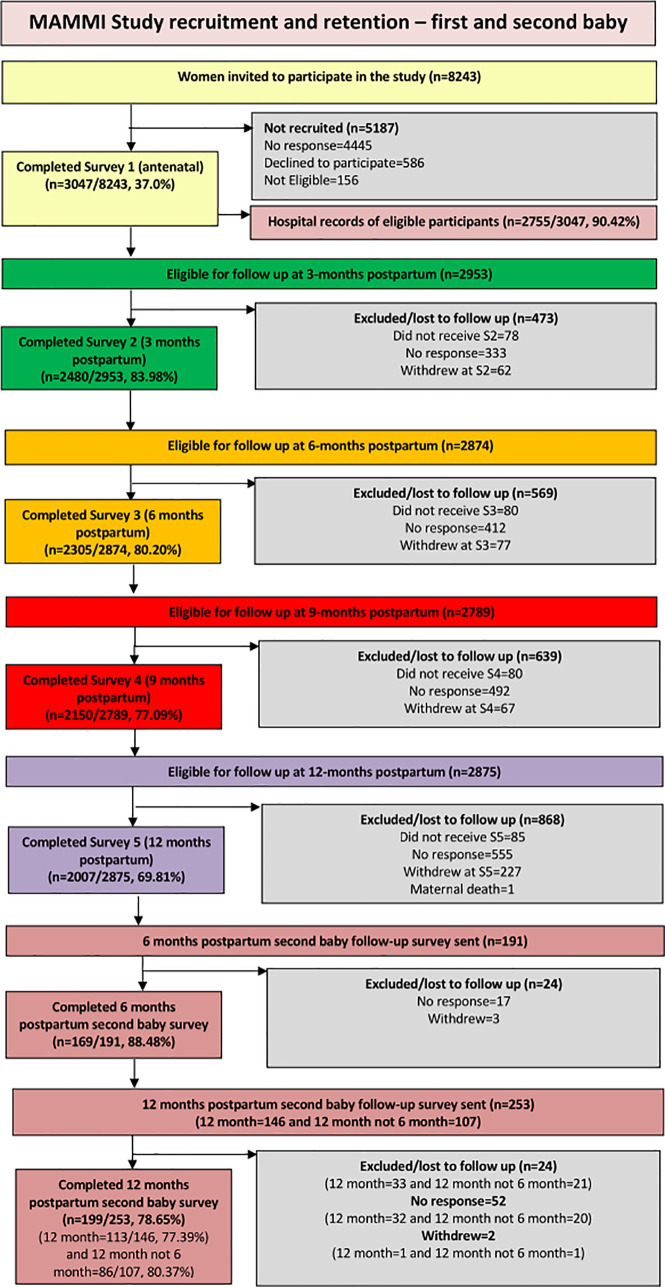
Flow chart for participation rates for MAMMI and SIM.

Participants completed a self-administered survey in early pregnancy, and at 3, 6, 9, and 12-months postpartum. The first survey included retrospective questions concerning their health before and during pregnancy, and the 3-month postpartum survey included retrospective questions about their health at the end of pregnancy and about the birth. After women completed the 12-month postpartum survey, all eligible participants who had consented to being contacted about taking part in related research were asked if they were willing to opt in to the second baby follow-up study. Participants were eligible if they had had their second baby in the year before the survey’s administration. A total of 254 women who completed an additional survey 6 and/or 12-months after their second baby birth were included as part of the Second baby, Intervention and Measuring Cost (SIM) study, (see Fig 1).

Because participation required multiple follow-up surveys several years, there is the potential for selection bias toward women with higher educational attainment and stable social circumstances. Although this convenience sample may limit generalisability, particularly to more socioeconomically disadvantaged populations, the large sample size, broad inclusion criteria (no restrictions on medical or obstetric risk factors), and stratification by the existence of the morbidities before the woman’s first pregnancy, help mitigate this bias and provide valuable insights into morbidity trajectories in a well-characterised cohort of women.

The sample size of the initial (first baby) MAMMI study was powered to ensure inclusion of 240 women with UI to detect associations between UI postpartum and maternal age, ethnicity, and pre-pregnancy body mass index (BMI), with the criterion for significance set at p = < 0.05. As there were no data on UI in an Irish population of peripartum women at the time of starting the study, the sample size was calculated on the proportion of women affected by UI based on findings from three international studies [40–42]. The data collected in the antenatal survey and all the four surveys up to 12- months postpartum were self-reported. Data on women’s first labour and birth outcomes were collected in the 3-month postpartum survey and collected from consenting women’s maternity hospital records. The data collected in the MAMMI-SIM study, including data on second and subsequent births, were self-reported.

In terms of recall bias, the accuracy of women’s memories of their experiences, and their agreement with data recorded in their maternity care records [43], even up to five years after the birth, has been documented [44].

All women who completed the MAMMI study, consented to being re-contacted about related research, and had a second birth during the SIM study were invited to complete two additional surveys at **both** 6 and 12-months postpartum after their second birth. In practice, not every woman returned both surveys: some completed only the 6-month, some only the 12-month, and others both. In order for their data to be eligible for inclusion in this study on persistence of morbidities, and to avoid potential non-response bias, the sample was restricted to only those women who had completed all the relevant surveys (6 and 12-months after the birth of the first child, and 6 and 12-months after the birth of the second child).

This means, for example:

If a woman completed the 6-month survey after her first baby’s birth but not the 12-month survey, **and** completed only the 12-month survey after her second baby’s birth but not the 6-month survey, her data were ineligible for inclusion because persistence of a morbidity could not be definitively identified.

### 2.3 Survey measures

Respondents were asked to give information on their experiences of the six previously outlined morbidities: urinary incontinence, faecal incontinence, pelvic girdle pain, sexual health problems, depression and anxiety.

*Urinary incontinence* (UI) was defined as reporting any leakage of urine when coughing, laughing, sneezing or exercising, when leaking on the way to the toilet, when having to wait to use the toilet, or when not going to the toilet immediately [45]. The morbidity was deemed present if the respondent said they experienced any of these elements of the condition “one or several times a month”.

*Faecal incontinence* (FI) was defined as reporting any leakage of liquid or solid stool [46]. Similar to UI, the morbidity was considered present if the respondent experienced one or both of these symptoms “one or several times a month”.

*Any sexual health problem* (SHP) was defined as women reporting any of the following problems: dyspareunia, low libido and/or vaginal dryness [47].

*Pelvic girdle pain* (PGP) was measured using a labeled pain diagram and defined as the presence of any pain between the posterior iliac crest and the inferior gluteal fold or at the pubic symphysis.

*Depression* and *anxiety* were measured at each time point during pregnancy and postpartum using the Depression, Anxiety, Stress Scale (DASS), which asks about symptoms in the past week [48].

The short-form Depression, Anxiety and Stress Scale (DASS 21) [49,50] was used to assess prevalence and change in mental health symptoms relating to depression, anxiety and stress from pregnancy through the first postpartum year. The DASS-21 contains three sub-scales that consist of seven items each. Responses are measured on a four-point Likert scale from ‘*Did not apply to me at all’* to ‘*Applied to me very much or most of the time*’. The Depression sub-scale includes statements aimed to detect hopelessness, self-deprecation, devaluation of life and dysphoria (distress/discomfort). The Anxiety sub-scale includes statements concerning autonomic arousal (physical sensations of anxiety such as heart palpations or dry mouth) and situational anxiety. The Stress sub-scale includes statements relating to difficulties with relaxing, and threshold for agitation.

For all six morbidities, survey 1 (completed during the first pregnancy at approximately 10-18 weeks’ gestation) asked about symptoms in the 12-months before and since the start of pregnancy, and all postnatal surveys, at 3, 6, 9 and 12-months postpartum asked about symptoms in the past three months.

### 2.4 Data analysis

Participants’ data were first analysed across a range of characteristics/factors gathered in the first survey to assess whether significant differences existed between the participants in subsequent survey waves. Age, BMI and educational status were compared between those who participated in the MAMMI study only and those who also participated in the SIM study. Chi-squared testing was used to determine any statistically significant differences in characteristics between groups.

Next, the prevalence of each of the six morbidities (UI, FI, PGP, SHP, depression, anxiety) was measured at all survey time points. Women were initially stratified into two groups, those who did and those who did not have the morbidity in the 12-months before their first pregnancy. Subsequent analyses measured the positive prevalence of each morbidity at each survey time point.

Persistence of each morbidity, as previously defined, was assessed amongst the two stratified groups. A condition was considered non-persistent if it was experienced after the first baby but not after the second baby. To limit any potential non-response bias, the sample was restricted to only those women who completed all the relevant surveys (6 and 12- months after the first baby’s birth, and 6 and 12-months after the second baby’s birth). To determine statistically significant differences (p < 0.05) we calculated relative risk ratios (RR) with 95% confidence intervals (CI).

In this study, partially completed surveys were defined as those in which participants responded to some, but not necessarily all, questions on each morbidity outcome. Surveys were included in the analysis if participants provided complete responses for the specific morbidity-related items being analysed at each time point. Surveys with no responses on a morbidity were excluded from the analysis for that particular morbidity and time point. Consequently, the sample size for analyses varied slightly across outcomes and time points, which is clearly reported within each relevant results table.

All statistical analyses were conducted using Stata version 16 (StataCorp LLC, College Station, TX, USA).

## 3. Results

### 3.1 Participant characteristics

The study participants’ characteristics (measured in the first MAMMI study survey that was completed during early pregnancy with their first child) are presented in Table 1. Data are stratified in three ways: (a) the characteristics of all participants who were included in the first baby surveys (MAMMI); (b) the characteristics of only the women who participated in both the first and second baby surveys (SIM); and (c) the characteristics of women who completed the first, but not second baby, surveys (MAMMI, but not SIM). Statistically significant differences were seen in age, educational level, relationship and employment status (p<0.05) between those in the MAMMI only study and those who also completed SIM study.

**Table 1:** Participant characteristics for MAMMI and SIM.

	(a) MAMMI (n = 3,047)	(b)SIM (n = 254)	(c)MAMMI but not SIM (n = 2,793)	Chi square tests of independence (between (b) and 3 (c))
Age Groups*				
Up to 24	239 (7.8%)	6 (2.4%)	233 (8.3%)	χ2$\chi^{\text{2}}$ (4) = 29.06
25-29	620 (20.4%)	37 (14.6%)	583 (20.9%)	*p < 0.01*
30-34	1,316 (43.2%)	130 (51.2%)	1,186 (42.5%)	
35-39	734 (24.1%)	77 (30.3%)	657 (23.5%)	
40 and over	138 (4.5%)	4 (1.6%)	134 (4.8%)	
BMI**				
Underweight (<18.5)	141 (5.0%)	17 (7%)	124 (4.8%)	χ2$\chi^{\text{2}}$ (5) = 3.74
Normal (18.5–24.9)	1,833 (65.4%)	150 (61.7%)	1,683 (65.7%)	*p = 0.587*
Overweight (25.0–29.9)	540 (19.3%)	50 (20.6%)	490 (19.1%)	
Moderately obese (30–39.9)	237 (8.5%)	23 (9.5%)	214 (8.4%)	
Extremely obese (>40)	51 (1.8%)	3 (1.2%)	48 (1.9%)	
**Education Status**				
Junior Cert or less	70 (2.3%)	1 (0.40%)	69 (2.5%)	χ2$\chi^{\text{2}}$ (3) = 26.29
Leaving Cert/Vocational	896 (29.7%)	44 (17.5%)	852 (30.8%)	*p < 0.01*
Primary degree/PGcert	1,365 (45.2%)	139 (55.2%)	1,226 (44.3%)	
MSc/PhD	690 (22.8%)	68 (27%)	622 (22.5%)	
**Relationship status**				
Cohabiting partner	2,672 (88.1%)	243 (95.7%)	2,429 (87.4%)	$\chi^{\text{2}}$ (3) = 15.45
Non-cohabiting partner	236 (7.8%)	6 (2.4%)	230 (8.3%)	*p < 0.01*
Single	101 (3.3%)	4 (1.6%)	97 (3.5%)	
Other	25 (0.8%)	1 (0.4%)	24 (0.9%)	
**Employment status**				
Full time employment	2,412 (79.3%)	228 (89.8%)	2,184 (78.4%)	$\chi^{\text{2}}$ (5) = 23.04
Part time employment	253 (8.3%)	12 (4.7%)	241 (8.7%)	*p < 0.01*
Not in paid employment	243 (8.0%)	4 (1.6%)	239 (8.6%)	
Student	52 (1.7%)	5 (2.0%)	47 (1.7%)	
Sick or disabled	21 (0.7%)	1 (0.4%)	20 (0.7%)	
Other	59 (1.9%)	4 (1.6%)	55 (2.0%)	
**Accommodation status**				
Home, no mortgage	238 (7.9%)	16 (6.3%)	22 (8%)	χ2$\chi^{\text{2}}$ (6) = 11.88
Home, mortgage	1,524 (50.3%)	148 (58.3%)	1,376 (49.5%)	*p = 0.065*
Home, private rental	1,080 (35.6%)	84 (33.1%)	996 (35.9%)	
Home, Local Authority	107 (3.5%)	4 (1.6%)	103 (3.7%)	
Caravan/Mobile Home	10 (0.3%)	–	10 (0.4%)	
Hostel/Homeless	5 (0.2%)	4 (1.6%)	5 (0.2%)	
Other	68 (2.2%)	2 (0.8%)	66 (2.4%)	

**MAMMI** refers to those who enrolled in the study for their first baby. **SIM** refers to those who enrolled in the study for their second baby, having already participated in the MAMMI study. **MAMMI but not SIM** refers to those who enrolled in the study for their first baby but did not participate in the SIM study.

*Age group formation in line with standard data collection for the study of pregnant women by institutions such as the National Center for Health Statistics [51].

**BMI categories as per the Health Service Executive [52].

#### a. Prevalence of morbidity during the first pregnancy and after the first and second birth.

The respondents were stratified into two groups, those who did and did not experience the morbidity of interest in the 12-months before their first pregnancy. The prevalence of each condition (UI, FI, PGP, sexual health problems, depression and anxiety) was then measured in both groups (see Fig 1 and S1 Table and S2 Table for detailed results).

Higher prevalence was seen consistently across all six morbidities among those who had experienced the condition in 12-months before their first pregnancy. For those who had experienced the morbidity in 12-months before their first pregnancy (i.e., those represented by the red trend line in Fig 1), the 12-month postpartum prevalence was lower than the prevalence measured during pregnancy, the exception being prevalence of faecal incontinence (full details in S1 Table). However, those who had not experienced the morbidity in 12-months before their first pregnancy (i.e., those represented by the blue trend line in Fig 1) showed a higher prevalence 12-month postpartum for urinary incontinence, pelvic girdle pain and depression (full details in S2 Table).

#### b. Persistence of morbidity after having a first and second child.

Table 2 outlines the six identified morbidities, stratified by whether the participant had experienced the morbidity in 12-months before their first pregnancy. A statistically significant difference, as per relative risk ratio (p < 0.05), was found between these two groups across four of those morbidities: UI, SHP, depression and anxiety.

**Table 2:** Persistence of morbidity (morbidity experienced 6-12 months after first baby and 6-12 months after second baby) – Total eligible sample (n = 254).

	Did experience persistence	Did not experience persistence	Relative Risk ratio (95%CI)	Fisher’s Exact Test
**Urinary Incontinence (n = 91)**			*2.53 (1.95-3.29)*	*p < 0.05*
Did have the morbidity in the 12-months before the first pregnancy (n = 5)	5 (100%)	0		
Did **not** have the morbidity in the 12-months before the first pregnancy (n = 86)	34 (39.53%)	52 (60.47%)		
**Faecal Incontinence (n = 100)**			–	–
Did have the morbidity in the 12-months before the first pregnancy (n = 0)	–	–		
Did **not** have the morbidity in the 12-months before the first pregnancy (n = 100)	–	100 (100%)		
**Pelvic Girdle Pain (n = 86)**			*1.01 (0.94-1.09)*	*p > 0.05*
Did have the morbidity in the 12-months before the first pregnancy (n = 53)	52 (98.11%)	1 (1.89%)		
Did **not** have the morbidity in the 12-months before the first pregnancy (n = 33)	32 (96.97%)	1 (3.03%)		
**Sexual health problems (n = 124)**			*1.12 (1.02-1.23)*	*p < 0.05*
Did have the morbidity in the 12-months before the first pregnancy (n = 76)	76 (100%)	0		
Did **not** have the morbidity in the 12-months before the first pregnancy (n = 48)	43 (89.58%)	5 (10.42%)		
**Depression (n = 87)**			*2.63 (1.15-6.03)*	*p = −0.065*
Did have the morbidity in the 12-months before the first pregnancy (n = 8)	4 (50%)	4 (50%)		
Did **not** have the morbidity in the 12-months before the first pregnancy (n = 79)	15 (18.99%)	64 (81.01%)		
**Anxiety (n = 90)**			*7.42 (4.38-12.55)*	*p > 0.05*
Did have the morbidity in the 12-months before the first pregnancy (n = 1)	1 (100%)	0 (0%)		
Did **not** have the morbidity in the 12-months before the first pregnancy (n = 89)	12 (13.48%)	77 (86.52%)		

## 4. Discussion

### Individual morbidities

#### Urinary incontinence (UI).

The prevalence of UI was especially high in women with a history of UI in the 12-months before their first pregnancy. This is in line with Patel et al. [53] who found that the strongest predictor of UI during pregnancy was UI before pregnancy, and the strongest predictors of postpartum UI were UI before pregnancy and during pregnancy. In their study with 3,001 participants, the prevalence of UI among first-time mothers increased from 12.5% at 6-months postpartum to 27.4% at 30-months postpartum, and 52.1% reported UI at one or more postpartum data collection stages. In our study, the prevalence of UI did not decrease postpartum for women with a history of UI in the 12-months before their first pregnancy, and the prevalence remained high after their second baby. For women without a history of UI, there was a prevalence peak in the first three months postpartum, then it dropped slightly before plateauing at a higher prevalence than during pregnancy. This demonstrates that some women without any UI in the 12-months before their first pregnancy will develop persistent UI subsequently. The UI prevalence 12-months after having had a second baby was higher in both groups (with or without UI in the 12-months before their first pregnancy). The fact that study participants had to opt in for the follow-up study after the second baby might have inflated the prevalence if more women with existing health problems chose to take part. A systematic review [54] on the association between parity and UI during pregnancy and the first year postpartum found that women who had already had one or more child(ren) were more likely to report UI at some point during their pregnancy but not when UI was measured over a 4-week period. At three months postpartum UI was also associated with multiparity. While these results were based on data from 39 studies, the evidence was uncertain. Barbosa et al.[55] also found very low quality of evidence from a systematic review of 13 studies and showed that multiparity was a risk factor for UI in pregnancy. However, the authors did not examine UI postpartum.

MacArthur et al., explored health women’s problems up to 6 years postpartum and found that increasing parity was associated with persistent UI [56]. Our findings show a statistically significant difference in persistence between those that did experience urinary incontinence in the 12-months before their first pregnancy and those that did not (RR 2.53, 95% CI (1.95–3.29)).

#### Faecal incontinence (FI).

The prevalence of FI decreased one year after the first baby’s birth in women with a history of FI in the 12-months before their first pregnancy but not in women with no prior history, although the prevalence was lower in the latter group. The prevalence in both groups remained low after the birth of the second baby. Of the 100 complete responses, none reported FI in the 12-months before their first pregnancy and when followed up in the SIM study reported no persistence of FI at 6 or 12-months postpartum.

Gartland et al.[57] also found that there was no association between parity and UI or FI reported at four years postpartum. Webb et al.[58] did find that, in 175 women who experienced Obstetric Anal Sphincter Injury (OASI) in a previous pregnancy, the odds of reporting poor quality of life (QoL) for ‘incontinence impact’, measured using the Manchester Health Questionnaire (MHQ) scale, were significantly higher for women who had three or more children compared with women after their second baby’s birth. A large longitudinal study [59] following women up to 12 years after the index birth found that 6% (n=227/3,763) had persistent FI and 42.7% of those who reported FI 3-months after the index pregnancy also reported it at 12 years. The same pattern of persistence and resolution of FI was evident for women of any parity at the index birth and separately for those who had just birthed their first baby. In their study, the prevalence of FI was 4.0% after the first birth and 6.0% after a second birth AOR 1.50 [95% CI 0.88–2.57, p=0.135].

#### Pelvic girdle pain (PGP).

The prevalence of PGP was high at all follow-up time points, particularly in women with a history of PGP in the 12-months before their first pregnancy. Interestingly, PGP is commonly considered to resolve after the birth [19]. Contrary to this perception, many women in our study continued to report PGP symptoms postpartum after the first and second baby. In women with a history of in the 12-months before their first pregnancy the prevalence peaked during pregnancy and dropped to some extend postpartum, but about eight in ten still reported PGP at some point between 9 and 12-months after their first and second baby. Consistent with the high prevalence findings in other studies [17,60], nearly half of women without a history of PGP in the 12-months before their first pregnancy experienced PGP during their first pregnancy. The prevalence in this group increased postpartum and remained high in the 12-months after their first baby’s birth and after their second baby’s birth. These findings indicate the importance of exploring subgroups of PGP. Persistence of PGP was not associated with having a history of PGP in the 12-months before their first pregnancy (RR 1.01, 95% CI (0.94-1.09)). A recent scoping review on PGP risk factors reported that most existing studies found that parity and having had PGP in a previous pregnancy were risk factors for PGP during pregnancy [61]. PGP during pregnancy is a risk factor for PGP postpartum [62], but it is unclear how having PGP postpartum after the first baby’s birth might impact on postpartum symptoms following the second or subsequent births.

To some extent, such high prevalence may be due to the broad definition (any self-reported pain on a diagram) we used in this study and because we examined period prevalence. Nevertheless, this study gives unique insights into PGP symptoms across pregnancy and the postpartum period after the first and second baby through its stratification by a history of PGP in the 12-months before their first pregnancy. Future research should conduct detailed examination of the potential trajectories of PGP before, during and after pregnancy, and explore the risk of PGP throughout a woman’s lifetime and how certain lifetime events such as pregnancy impact on these trajectories.

#### Sexual health problems.

Within this analysis, a high prevalence of SHP was seen, particularly amongst participants who had experienced problems in the 12-months before their first pregnancy. A fall in prevalence rates was seen across both studies from 3 months postpartum onwards. The prevalence of SHP was significantly higher at 6 and 12-months after their second baby’s birth in those who had experienced problems in the 12-months before their first pregnancy compared with those who did not and show a statistically significant difference in persistence (RR 1.12, 95% CI (1.02–1.23)).

The causes of SHP vary amongst women [63]. Mode of birth has been highlighted as an important risk factor with spontaneous vaginal births resulting in fewer postpartum problems in regard to sexual function [64]. Whilst childbirth has been found to have a lasting impact on sexual function, it may be more due to psychological rather than physical factors [65]. This will be explored further in the discussion of depression and anxiety.

Qualitative findings also suggest that when women seek professional support for these problems they are often met with unhelpful responses which discourage further engagement and lead to information being sought from other women [66]. A 2015 analysis of 1,507 first-time mothers within the Australian Maternal Health Study (MHS) found 89% of women reported SHP in the first 3-months postpartum. Despite the high prevalence, only 24% of participants recalled being asked about SHP by general practitioners (GP) and 14% by maternal and child health nurses, despite having contact with primary care practitioners during the first 3-months postpartum [67]. A separate study with women in the MAMMI study found that, of the women who experienced SHP after their first baby’s birth, only 2.9% (n = 18) experiencing dyspareunia and 3.6% (n = 16) experiencing a lack of vaginal lubrication spoke to their GP about these issues at 3-months postpartum [66].

An earlier 2018 study with the MAMMI study participants found breastfeeding was associated with dissatisfaction with body image and SHP at 6-months postpartum. Experiencing dyspareunia in the 12-months before their first pregnancy was associated with SHP at 6-months postpartum and a lack of vaginal lubrication at 12-months postpartum [21]. A 2013 study conducted in the US found that women who were older (estimated effect −1.1, p = .01), suffered from depression (−13.3; p = .01) and breastfed exclusively (−16.5, p < .001) reported significantly poorer sexual satisfaction [68]. The impact of parity has been studied across a range of populations, with multiparous women generally reporting better sexual satisfaction [68,69].

#### Depression and anxiety.

Our analysis indicates that the prevalence of symptoms of both depression and anxiety (not categorised by severity) across the different survey time points was higher amongst participants who had experienced symptoms in the 12-months before their first pregnancy (see Fig 1). While symptoms of depression fell at 12-months at the second baby’s birth, the prevalence of anxiety rose.

A statistically significant difference in the persistence of depression symptoms and anxiety symptoms after the second baby’s birth was associated with having had symptoms in the 12-months before their first pregnancy (depression RR 2.63, 95% CI (1.15–6.03); anxiety RR 7.42, 95% CI (4.38–12.55)).

Previous studies have highlighted the association between antenatal and postpartum depressive symptoms [70], with the onset of depressive symptoms mainly occurring in the early postpartum period and new onset symptoms accounting for half of these [71]. Across a range of studies one of the greatest predictors of postnatal depressive symptoms has been the occurrence of symptoms during pregnancy [72]. An earlier study amongst the MAMMI cohort found that depressive symptoms in pregnancy were associated with odds of 12.8 (95% CI 8.3–19.8) for postpartum depressive symptoms compared to women not reporting depressive symptoms in pregnancy [29]. However, other studies have found a decrease in minor depressive disorders during the postpartum period [73].

While multiparous women seem to have higher levels of certain risk factors for depressive and anxiety symptoms, specifically interpersonal risk [34], the impact of these risks may be reduced due to what has been described as their *more realistic* attitudes towards motherhood, particularly with regard to beliefs about maternal responsibility [34].

The consequences of depressive and anxiety symptoms can also impact other morbidities such as SHP. For example, women with depressive symptoms are less likely to have resumed intercourse at 6-months postpartum and more likely to report SHP than women without symptoms [74].

The continuation of these problems beyond the 6-week provision of postpartum maternity care services in Ireland has been identified as one justification for extending the period of postpartum care as part of an integrated system of care [28].

### Participant characteristics

When comparing the differences in participant characteristics between those who participated in the MAMMI study only and those who participated in both MAMMI and SIM we can see statistically significant differences in age, education, relationship and employment status (p < 0.05) (see Table 1), with the caveat that the participant numbers in SIM (n = 254) and MAMMI only (n = 2,793) are vastly different.

The age of those who participated in the SIM study tended to be slightly older than those who participated in MAMMI study only. For example, 51% of participants in the SIM study were aged 30–34 years when they had their first baby compared with 42% in MAMMI study.

The slightly younger age of MAMMI study only participants may reflect a subsequent delay in having their second baby. Data from the most recent Irish Census has shown that the number of women delaying pregnancy into their late 30s and 40s has increased [75].

The SIM study participants also saw a higher number with a primary degree/post graduate certificate or masters/PhD (a combined 82.2%) compared to MAMMI study only participants (66.8%). The higher levels of education associated with participation in both studies may represent an element of self-selection bias. Education and health status have been found to be crucial factors for taking part in other studies [76,77].

SIM study participants also had a higher level of full-time employment (89.8%) compared with MAMMI study only participants (78.4%). The discussion of employment uncertainty and its subsequent negative impact on fertility has been explored in several studies [78,79].

### Unmet health need

It is accepted that the scope of maternal health services must expand beyond those focused solely on pregnancy and childbirth. There is an increased acknowledgement that emphasis must continue to be placed on pre-pregnancy and long-term health [80]. The scope of what is considered within postpartum care must adopt a comprehensive approach including support regarding the physical recovery from childbirth, as well as the mental and emotional well-being of women as mothers [80]. The nature of longitudinal studies allows us to consider the persistence of certain health conditions. Understanding the scale of morbidities is crucial to ensure that any ongoing or unmet health needs are addressed. Further research is required to understand the health and help-seeking behaviour of peripartum women with health problems and explore if women understand what health issues are common, but not normal and amenable to remedial action.

#### c.Study strengths & limitations.

This study is the first in Ireland to consider the prevalence and persistence of key morbidities amongst women following the birth of their first baby, and to analyse the differences between women’s experience of these morbidities after their second baby’s birth.

**Fig 2:**
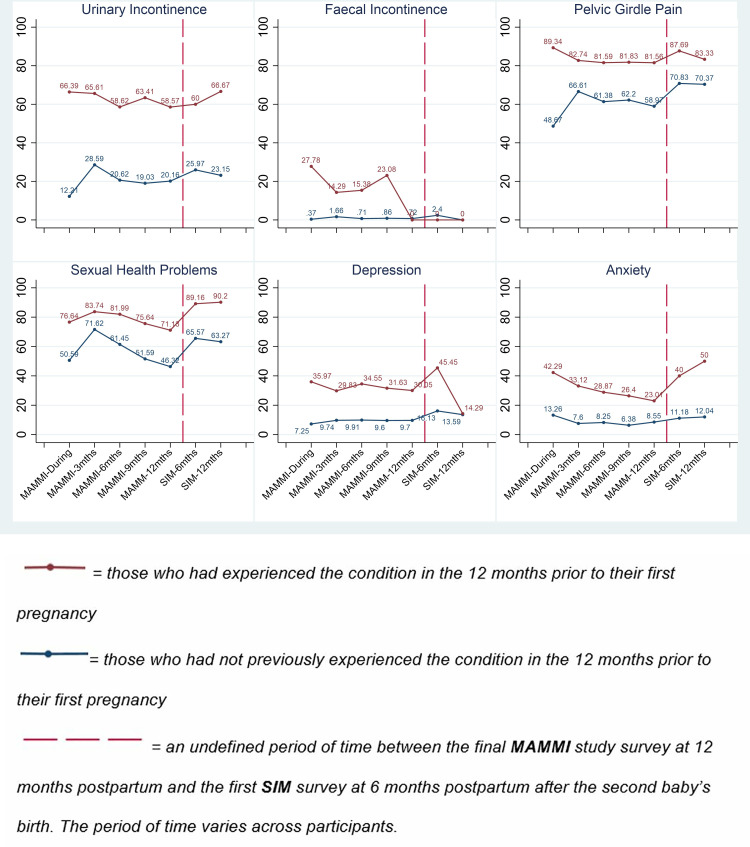
Prevalence (%) of morbidity amongst respondents of MAMMI and SIM surveys at each of the survey time points. a. Legend: Prevalence (%) of morbidity amongst respondents of MAMMI and SIM surveys at each of the survey time points.

One of the limitations of this study is the number of participants in the second baby follow-up was much lower (n = 254) than in the initial cohort (n = 3,047) (see Fig 2, Flowchart). Due to ethical considerations, women had to opt into this follow-up study, which contributed to the lower number since we were depended on women letting us know that they had had a second baby in the year before the study was conducted.

Several sources of bias may have influenced our findings. First, because participation in the SIM study was opt-in, women with persistent or more severe morbidities may have been likely to continue, potentially inflating prevalence and persistence estimates (self-selection bias). Second, our reliance on self-reported symptoms introduces recall and social-desirability biases; women may under- or over-report certain morbidities, particularly sensitive issues like UI, FI and SHP. Third, loss to follow-up and partially completed surveys, though explicitly handled as described in the methods section, could also skew results if non-responders differed systematically from responders. Finally, the convenience sampling at urban maternity hospitals, and the relatively high educational attainment in our cohort, may limit generalisability to more diverse populations.

Despite these limitations, the large sample size, prospective design, and stratification by women’s health history before their first pregnancy strengthen internal validity, and the biases described here should be addressed in future work employing probability sampling and objective clinical measures.

To validate and extend these findings, further studies using probability sampling and more diverse, representative populations are recommended. This will help ensure broader applicability of the results.

Future studies should consider performing multivariable analyses to gain a deeper understanding of the independent contribution of potential risk factors, such as age, BMI, mode of birth, education, employment, and pre-existing conditions, to the persistence and severity of maternal morbidities over successive pregnancies. A multivariable analysis approach would allow for more robust conclusions about causal relationships and could further inform targeted interventions in maternal and women’s healthcare.

## 5. Conclusion

This study offers critical insights into the prevalence and persistence of maternal morbidities following childbirth, emphasising the significant impact of pre-existing health conditions on postpartum health. Women with a history of UI experienced higher rates of persistence postpartum, reinforcing findings from previous research that underscore the predictive nature of symptoms that pre-exist a woman’s first pregnancy. The persistence of PGP and SHP further highlights the need for tailored management strategies in postpartum care, as many women report ongoing issues and unmet health needs. The elevated prevalence of depression and anxiety symptoms underscores the importance of integrating mental health support into maternal health services.

The persistence of maternal morbidities at 6 and 12-months postpartum, after a first and/or a second baby’s birth underscores the urgent need for enhanced access to longer-term healthcare to promote better maternal health and quality of life. Overall, these findings advocate for a comprehensive approach to postpartum care that extends beyond the 6-week postpartum period. This care needs to address the multifaceted needs of women, emphasise early identification, continuous support, and targeted interventions to improve maternal health outcomes.

## Supporting information

S1 Table Prevalence of morbidity amongst respondents of MAMMI and SIM surveys if they did report experiencing the condition in the 12-months prior to the pregnancy of their first child. (DOCX)

S2 Table Prevalence of morbidity amongst respondents of MAMMI and SIM surveys if they did not experience the condition in the 12-months prior to the pregnancy of their first child. (DOCX)

## References

[1] Knaul FM, Langer A, Atun R, Rodin D, Frenk J, Bonita R. Rethinking maternal health. Lancet Glob Health. 2016;4(4):e227-8. doi: 10.1016/S2214-109X(16)00044-9 26953968

[2] Women’s health and well-being in Europe: beyond the mortality advantage. Copenhagen: World Health Organization, Regional office for Europe; 2016.

[3] Institute for Health Metrics and Evaluation (IHME). GBD Compare Data Visualization. 2020. Available: http://vizhub.healthdata.org/gbd-compare

[4] Countdown to 2030 Collaboration. Countdown to 2030: tracking progress towards universal coverage for reproductive, maternal, newborn, and child health. Lancet. 2018;391(10129):1538–48. doi: 10.1016/S0140-6736(18)30104-1 29395268

[5] Koblinsky M, Chowdhury ME, Moran A, Ronsmans C. Maternal morbidity and disability and their consequences: neglected agenda in maternal health. J Health Popul Nutr. 2012;30(2):124–30. doi: 10.3329/jhpn.v30i2.11294 22838155 PMC3397324

[6] Gon G, Leite A, Calvert C, Woodd S, Graham WJ, Filippi V. The frequency of maternal morbidity: A systematic review of systematic reviews. Int J Gynaecol Obstet. 2018;141 Suppl 1(Suppl Suppl 1):20–38. doi: 10.1002/ijgo.12468 29851116 PMC6001670

[7] Haylen BT, de Ridder D, Freeman RM, Swift SE, Berghmans B, Lee J, et al. An International Urogynecological Association (IUGA)/International Continence Society (ICS) joint report on the terminology for female pelvic floor dysfunction. Int Urogynecol J. 2010;21(1):5–26. doi: 10.1007/s00192-009-0976-9 19937315

[8] Daly D, Clarke M, Begley C. Urinary incontinence in nulliparous women before and during pregnancy: prevalence, incidence, type, and risk factors. Int Urogynecol J. 2018;29(3):353–62. doi: 10.1007/s00192-018-3554-1 29362836

[9] Moossdorff-Steinhauser HFA, Berghmans BCM, Spaanderman MEA, Bols EMJ. Prevalence, incidence and bothersomeness of urinary incontinence between 6 weeks and 1 year post-partum: a systematic review and meta-analysis. Int Urogynecology J. 2021;32: 1675–93. doi: 10.1007/s00192-021-04877-w PMC8295150 34142179

[10] Abrams P, Andersson K-E, Apostolidis A, Birder L, Bliss D, Brubaker L, et al. 6th International Consultation on Incontinence. Recommendations of the International Scientific Committee: evaluation and treatment of urinary incontinence, pelvic organ prolapse and faecal incontinence. Neurourol Urodyn. 2018;37(7):2271–2. doi: 10.1002/nau.23551 30106223

[11] Brown SJ, Donath S, MacArthur C, McDonald EA, Krastev AH. Urinary incontinence in nulliparous women before and during pregnancy: prevalence, incidence, and associated risk factors. Int Urogynecol J. 2010;21(2):193–202. doi: 10.1007/s00192-009-1011-x 19834637

[12] Hannestad YS, Rortveit G, Sandvik H, Hunskaar S, Norwegian EPINCONT study. Epidemiology of Incontinence in the County of Nord-Trøndelag. A community-based epidemiological survey of female urinary incontinence: the Norwegian EPINCONT study. Epidemiology of Incontinence in the County of Nord-Trøndelag. J Clin Epidemiol. 2000;53(11):1150–7. doi: 10.1016/s0895-4356(00)00232-8 11106889

[13] Everist R, Burrell M, Mallitt K-A, Parkin K, Patton V, Karantanis E. Postpartum anal incontinence in women with and without obstetric anal sphincter injuries. Int Urogynecol J. 2020;31(11):2269–75. doi: 10.1007/s00192-020-04267-8 32157322

[14] Hage-Fransen MAH, Wiezer M, Otto A, Wieffer-Platvoet MS, Slotman MH, Nijhuis-van der Sanden MWG, et al. Pregnancy- and obstetric-related risk factors for urinary incontinence, fecal incontinence, or pelvic organ prolapse later in life: A systematic review and meta-analysis. Acta Obstet Gynecol Scand. 2021;100(3):373–82. doi: 10.1111/aogs.14027 33064839

[15] Albert H, Godskesen M, Westergaard J. Prognosis in four syndromes of pregnancy-related pelvic pain. Acta Obstet Gynecol Scand. 2001;80(6):505–10. 11380285

[16] Robinson HS, Mengshoel AM, Veierød MB, Vøllestad N. Pelvic girdle pain: potential risk factors in pregnancy in relation to disability and pain intensity three months postpartum. Man Ther. 2010;15(6):522–8. doi: 10.1016/j.math.2010.05.007 20621546

[17] Kovacs FM, Garcia E, Royuela A, González L, Abraira V, Spanish Back Pain Research Network. Prevalence and factors associated with low back pain and pelvic girdle pain during pregnancy: a multicenter study conducted in the Spanish National Health Service. Spine (Phila Pa 1976). 2012;37(17):1516–33. doi: 10.1097/BRS.0b013e31824dcb74 22333958

[18] Mackenzie J, Murray E, Lusher J. Women’s experiences of pregnancy related pelvic girdle pain: A systematic review. Midwifery. 2018;56:102–11. doi: 10.1016/j.midw.2017.10.011 29096278

[19] Wuytack F, Curtis E, Begley C. Experiences of First-Time Mothers With Persistent Pelvic Girdle Pain After Childbirth: Descriptive Qualitative Study. Phys Ther. 2015;95(10):1354–64. doi: 10.2522/ptj.20150088 25929535

[20] Srisopa P, Lucas R. Women’s Experience of Pelvic Girdle Pain After Childbirth: A Meta-Synthesis. J Midwifery Womens Health. 2021;66(2):240–8. doi: 10.1111/jmwh.13167 33314586

[21] O’Malley D, Higgins A, Begley C, Daly D, Smith V. Prevalence of and risk factors associated with sexual health issues in primiparous women at 6 and 12 months postpartum; a longitudinal prospective cohort study (the MAMMI study). BMC Pregnancy Childbirth. 2018;18: 196. doi: 10.1186/s12884-018-1838-6 29855357 PMC5984394

[22] Gommesen D, Nøhr E, Qvist N, Rasch V. Obstetric perineal tears, sexual function and dyspareunia among primiparous women 12 months postpartum: a prospective cohort study. BMJ Open. 2019;9(12):e032368. doi: 10.1136/bmjopen-2019-032368 31848167 PMC6937116

[23] Cattani L, De Maeyer L, Verbakel JY, Bosteels J, Deprest J. Predictors for sexual dysfunction in the first year postpartum: A systematic review and meta‐analysis. BJOG Int J Obstet Gynaecol. 2022;129: 1017–28. doi: 10.1111/1471-0528.16934 34536325

[24] Wang L, Wu T, Anderson JL, Florence JE. Prevalence and risk factors of maternal depression during the first three years of child rearing. J Womens Health (Larchmt). 2011;20(5):711–8. doi: 10.1089/jwh.2010.2232 21426237

[25] Ahmed A, Bowen A, Feng CX, Muhajarine N. Trajectories of maternal depressive and anxiety symptoms from pregnancy to five years postpartum and their prenatal predictors. BMC Pregnancy Childbirth. 2019;19(1):26. doi: 10.1186/s12884-019-2177-y 30642277 PMC6332639

[26] Shorey S, Chee CYI, Ng ED, Chan YH, Tam WWS, Chong YS. Prevalence and incidence of postpartum depression among healthy mothers: A systematic review and meta-analysis. J Psychiatr Res. 2018;104:235–48. doi: 10.1016/j.jpsychires.2018.08.001 30114665

[27] Jones K, Folliard K, Di Malta G, Oates J, Gilbert L, Harrison V. Risk factors associated with postpartum anxiety in Australia, Europe, and North America: A systematic review and narrative synthesis. J Affect Disord. 2025;373:478–94. doi: 10.1016/j.jad.2024.12.043 39778747

[28] Hannon S, Gartland D, Higgins A, Brown SJ, Carroll M, Begley C, et al. Physical health and comorbid anxiety and depression across the first year postpartum in Ireland (MAMMI study): A longitudinal population-based study. J Affect Disord. 2023;328:228–37. doi: 10.1016/j.jad.2023.02.056 36801420

[29] Hannon S, Gartland D, Higgins A, Brown SJ, Carroll M, Begley C, et al. Maternal mental health in the first year postpartum in a large Irish population cohort: the MAMMI study. Arch Womens Ment Health. 2022;25(3):641–53. doi: 10.1007/s00737-022-01231-x 35488067 PMC9072451

[30] MacArthur C, Wilson D, Herbison P, Lancashire RJ, Hagen S, Toozs-Hobson P, et al. Urinary incontinence persisting after childbirth: extent, delivery history, and effects in a 12-year longitudinal cohort study. BJOG. 2016;123(6):1022–9. doi: 10.1111/1471-0528.13395 25846816

[31] Bjelland EK, Eskild A, Johansen R, Eberhard-Gran M. Pelvic girdle pain in pregnancy: the impact of parity. Am J Obstet Gynecol. 2010;203(2):146.e1-6. doi: 10.1016/j.ajog.2010.03.040 20510180

[32] Saurel-Cubizolles MJ, Romito P, Lelong N, Ancel PY. Women’s health after childbirth: a longitudinal study in France and Italy. BJOG. 2000;107(10):1202–9. doi: 10.1111/j.1471-0528.2000.tb11608.x 11028569

[33] Dol J, Richardson B, Grant A, Aston M, McMillan D, Tomblin Murphy G, et al. Influence of parity and infant age on maternal self-efficacy, social support, postpartum anxiety, and postpartum depression in the first six months in the Maritime Provinces, Canada. Birth. 2021;48(3):438–47. doi: 10.1111/birt.12553 34008241

[34] Sockol LE, Battle CL. Maternal attitudes, depression, and anxiety in pregnant and postpartum multiparous women. Arch Womens Ment Health. 2015;18(4):585–93. doi: 10.1007/s00737-015-0511-6 25712795

[35] Fritel X, Gachon B, Saurel-Cubizolles MJ, EDEN Mother-Child Cohort Study Group. Postpartum psychological distress associated with anal incontinence in the EDEN mother-child cohort. BJOG. 2020;127(5):619–27. doi: 10.1111/1471-0528.16075 31872546

[36] O’Malley D, Smith V, Higgins A. Sexual Aspects of Problems in the Postpartum and Early Parenthood (1st Year). Midwifery and Sexuality. Springer International Publishing. 2023. p. 163–73. doi: 10.1007/978-3-031-18432-1_14

[37] Fitzgerald L, McNab S, Njau P, Chandra P, Koyiet P, Levine R, et al. Beyond survival: Prioritizing the unmet mental health needs of pregnant and postpartum women and their caregivers. PLOS Glob Public Health. 2024;4(2):e0002782. doi: 10.1371/journal.pgph.0002782 38315641 PMC10843059

[38] HPO (Health Pricing Office). Perinatal Statistics report 2012. 2013. Available: https://www.hpo.ie/latest_hipe_nprs_reports/NPRS_2012/Perinatal_Statistics_Report_2012.pdf

[39] HPO (Health Pricing Office). Perinatal Statistics report 2016. 2018. Available: https://www.hpo.ie/latest_hipe_nprs_reports/NPRS_2016/Perinatal_Statistics_Report_2016.pdf

[40] Brown S, Lumley J. Maternal health after childbirth: results of an Australian population based survey. Br J Obstet Gynaecol. 1998;105(2):156–61. doi: 10.1111/j.1471-0528.1998.tb10045.x 9501779

[41] Glazener CMA, Herbison GP, MacArthur C, Lancashire R, McGee MA, Grant AM, et al. New postnatal urinary incontinence: obstetric and other risk factors in primiparae. BJOG. 2006;113(2):208–17. doi: 10.1111/j.1471-0528.2005.00840.x 16412000

[42] Wesnes SL, Hunskaar S, Bo K, Rortveit G. The effect of urinary incontinence status during pregnancy and delivery mode on incontinence postpartum. A cohort study. BJOG. 2009;116(5):700–7. doi: 10.1111/j.1471-0528.2008.02107.x 19220234 PMC2675011

[43] Gartland D, Lansakara N, Flood M, Brown SJ. Assessing obstetric risk factors for maternal morbidity: congruity between medical records and mothers’ reports of obstetric exposures. Am J Obstet Gynecol. 2012;206(2):152.e1-10. doi: 10.1016/j.ajog.2011.10.863 22177183

[44] Takehara K, Noguchi M, Shimane T, Misago C. A longitudinal study of women’s memories of their childbirth experiences at five years postpartum. BMC Pregnancy Childbirth. 2014;14:221. doi: 10.1186/1471-2393-14-221 24996683 PMC4227007

[45] Haylen BT, de Ridder D, Freeman RM, Swift SE, Berghmans B, Lee J, et al. An International Urogynecological Association (IUGA)/International Continence Society (ICS) joint report on the terminology for female pelvic floor dysfunction. Neurourol Urodyn. 2010;29(1):4–20. doi: 10.1002/nau.20798 19941278

[46] Sultan AH, Monga A, Lee J, Emmanuel A, Norton C, Santoro G, et al. An International Urogynecological Association (IUGA)/International Continence Society (ICS) joint report on the terminology for female anorectal dysfunction. Int Urogynecol J. 2017;28(1):5–31. doi: 10.1007/s00192-016-3140-3 27774569

[47] Rogers RG, Pauls RN, Thakar R, Morin M, Kuhn A, Petri E, et al. An International Urogynecological Association (IUGA)/International Continence Society (ICS) joint report on the terminology for the assessment of sexual health of women with pelvic floor dysfunction. Neurourol Urodyn. 2018;37(4):1220–40. doi: 10.1002/nau.23508 29441607

[48] Lovibond S, Lovibond P. Manual for the Depression Anxiety Stress Scales. Sydney: Psychology Foundation; 1995.

[49] Lovibond PF, Lovibond SH. The structure of negative emotional states: comparison of the Depression Anxiety Stress Scales (DASS) with the Beck Depression and Anxiety Inventories. Behav Res Ther. 1995;33(3):335–43. doi: 10.1016/0005-7967(94)00075-u 7726811

[50] Henry JD, Crawford JR. The short-form version of the Depression Anxiety Stress Scales (DASS-21): construct validity and normative data in a large non-clinical sample. Br J Clin Psychol. 2005;44(Pt 2):227–39. doi: 10.1348/014466505X29657 16004657

[51] Woodall A, Driscoll AK. NCHS Data Brief: Racial and Ethnic Differences in Mortality Rates of Infants Born to Teen Mothers: United States, 2017-2018. 2020. Available: https://www.cdc.gov/nchs/data/databriefs/db371-h.pdf 33054910

[52] HSE. BMI Chart (Kgs/m2) for use with the Weight Management Treatment Algorithm. Health Service Executive; Available: https://www.hse.ie/eng/services/list/2/primarycare/east-coast-diabetes-service/management-of-type-2-diabetes/lifestyle-management/healthy-eating-advice/bmi-chart.pdf

[53] Patel K, Long JB, Boyd SS, Kjerulff KH. Natural history of urinary incontinence from first childbirth to 30-months postpartum. Arch Gynecol Obstet. 2021;304(3):713–24. doi: 10.1007/s00404-021-06134-3 34175975

[54] Wuytack F, Moran P, Daly D, Begley C. Is there an association between parity and urinary incontinence in women during pregnancy and the first year postpartum?: A systematic review and meta-analysis. Neurourol Urodyn. 2022;41(1):54–90. doi: 10.1002/nau.24785 34529861

[55] Barbosa L, Boaviagem A, Moretti E, Lemos A. Multiparity, age and overweight/obesity as risk factors for urinary incontinence in pregnancy: a systematic review and meta-analysis. Int Urogynecol J. 2018;29(10):1413–27. doi: 10.1007/s00192-018-3656-9 29754281

[56] MacArthur C, Glazener CMA, Wilson PD, Lancashire RJ, Herbison GP, Grant AM. Persistent urinary incontinence and delivery mode history: a six-year longitudinal study. BJOG. 2006;113(2):218–24. doi: 10.1111/j.1471-0528.2005.00818.x 16412001

[57] Gartland D, MacArthur C, Woolhouse H, McDonald E, Brown S. Frequency, severity and risk factors for urinary and faecal incontinence at 4 years postpartum: a prospective cohort. BJOG Int J Obstet Gynaecol. 2016;123: 1203–11. doi: 10.1111/1471-0528.13522 26179947

[58] Webb SS, Sitch A, MacArthur C. The impact of mode of subsequent birth after obstetric anal sphincter injury on bowel function and related quality of life: a cohort study. Int Urogynecol J. 2020;31(11):2237–45. doi: 10.1007/s00192-020-04234-3 32095959 PMC7561530

[59] MacArthur C, Wilson D, Herbison P, Lancashire RJ, Hagen S, Toozs-Hobson P, et al. Faecal incontinence persisting after childbirth: a 12 year longitudinal study. BJOG. 2013;120(2):169–79. doi: 10.1111/1471-0528.12039 23190303

[60] Ceprnja D, Chipchase L, Fahey P, Liamputtong P, Gupta A. Prevalence and Factors Associated with Pelvic Girdle Pain During Pregnancy in Australian Women: A Cross-Sectional Study. Spine (Phila Pa 1976). 2021;46(14):944–9. doi: 10.1097/BRS.0000000000003954 33492087 PMC8221721

[61] Wuytack F, Begley C, Daly D. Risk factors for pregnancy-related pelvic girdle pain: a scoping review. BMC Pregnancy Childbirth. 2020;20(1):739. doi: 10.1186/s12884-020-03442-5 33246422 PMC7694360

[62] Wiezer M, Hage-Fransen MAH, Otto A, Wieffer-Platvoet MS, Slotman MH, Nijhuis-van der Sanden MWG, et al. Risk factors for pelvic girdle pain postpartum and pregnancy related low back pain postpartum; a systematic review and meta-analysis. Musculoskelet Sci Pract. 2020;48:102154. doi: 10.1016/j.msksp.2020.102154 32560862

[63] Banaei M, Alidost F, Ghasemi E, Dashti S. A comparison of sexual function in primiparous and multiparous women. J Obstet Gynaecol. 2020;40(3):411–8. doi: 10.1080/01443615.2019.1640191 31537138

[64] Hicks TL, Goodall SF, Quattrone EM, Lydon-Rochelle MT. Postpartum sexual functioning and method of delivery: summary of the evidence. J Midwifery Womens Health. 2004;49(5):430–6. doi: 10.1016/j.jmwh.2004.04.007 15351333

[65] Botros SM, Abramov Y, Miller J-JR, Sand PK, Gandhi S, Nickolov A, et al. Effect of parity on sexual function: an identical twin study. Obstet Gynecol. 2006;107(4):765–70. doi: 10.1097/01.AOG.0000207677.03235.76 16582110

[66] O’Malley D, Smith V, Higgins A. Sexual health issues postpartum-A mixed methods study of women’s help-seeking behavior after the birth of their first baby. Midwifery. 2022;104:103196. doi: 10.1016/j.midw.2021.103196 34767981

[67] McDonald E, Woolhouse H, Brown SJ. Consultation about Sexual Health Issues in the Year after Childbirth: A Cohort Study. Birth. 2015;42(4):354–61. doi: 10.1111/birt.12193 26467855

[68] Yee LM, Kaimal AJ, Nakagawa S, Houston K, Kuppermann M. Predictors of postpartum sexual activity and function in a diverse population of women. J Midwifery Womens Health. 2013;58(6):654–61. doi: 10.1111/jmwh.12068 24325662 PMC4896481

[69] Martínez-Galiano JM, Hernández-Martínez A, Rodríguez-Almagro J, Delgado-Rodríguez M, Gómez-Salgado J. Relationship between parity and the problems that appear in the postpartum period. Sci Rep. 2019;9(1):11763. doi: 10.1038/s41598-019-47881-3 31409871 PMC6692385

[70] Andersson L, Sundström-Poromaa I, Wulff M, Aström M, Bixo M. Depression and anxiety during pregnancy and six months postpartum: a follow-up study. Acta Obstet Gynecol Scand. 2006;85(8):937–44. doi: 10.1080/00016340600697652 16862471

[71] Stowe ZN, Hostetter AL, Newport DJ. The onset of postpartum depression: Implications for clinical screening in obstetrical and primary care. Am J Obstet Gynecol. 2005;192(2):522–6. doi: 10.1016/j.ajog.2004.07.054 15695997

[72] Heron J, O’Connor TG, Evans J, Golding J, Glover V, ALSPAC Study Team. The course of anxiety and depression through pregnancy and the postpartum in a community sample. J Affect Disord. 2004;80(1):65–73. doi: 10.1016/j.jad.2003.08.004 15094259

[73] Josefsson A, Berg G, Nordin C, Sydsjö G. Prevalence of depressive symptoms in late pregnancy and postpartum. Acta Obstet Gynecol Scand. 2001;80(3):251–5. doi: 10.1034/j.1600-0412.2001.080003251.x 11207491

[74] Morof D, Barrett G, Peacock J, Victor CR, Manyonda I. Postnatal depression and sexual health after childbirth. Obstet Gynecol. 2003;102(6):1318–25. doi: 10.1016/j.obstetgynecol.2003.08.020 14662221

[75] McGlynn M. Women waiting until they are older to have babies. The Irish Examiner. 2022.

[76] Enzenbach C, Wicklein B, Wirkner K, Loeffler M. Evaluating selection bias in a population-based cohort study with low baseline participation: the LIFE-Adult-Study. BMC Med Res Methodol. 2019;19(1):135. doi: 10.1186/s12874-019-0779-8 31262266 PMC6604357

[77] Ögge LE, Murray F, Modzelewska D, Lundqvist R, Nilsson S, Carré H, et al. Maternal characteristics and pregnancy outcomes in the NICE birth cohort: an assessment of self-selection bias. J Matern Fetal Neonatal Med. 2022;35(25):9014–22. doi: 10.1080/14767058.2021.2011854 34979877

[78] Gatta A, Mattioli F, Mencarini L, Vignoli D. Employment uncertainty and fertility intentions: Stability or resilience?. Popul Stud (Camb). 2022;76(3):387–406. doi: 10.1080/00324728.2021.1939406 34468282 PMC9621103

[79] Hofmann B, Hohmeyer K. Perceived Economic Uncertainty and Fertility: Evidence From a Labor Market Reform. J of Marriage and Family. 2013;75(2):503–21. doi: 10.1111/jomf.12011

[80] Firoz T, McCaw-Binns A, Filippi V, Magee LA, Costa ML, Cecatti JG, et al. A framework for healthcare interventions to address maternal morbidity. Int J Gynaecol Obstet. 2018;141 Suppl 1(Suppl Suppl 1):61–8. doi: 10.1002/ijgo.12469 29851114 PMC6001624

