# Supplementary material for: The prevalence and persistence of maternal morbidities after first vs. second birth: A prospective cohort study in Ireland
Source: PLoS One. 2025 Oct 22;20(10):e0332891. doi: 10.1371/journal.pone.0332891 (PMC12543160; doi:10.1371/journal.pone.0332891)
Supplement: S1 Table — (DOCX) [file pone.0332891.s001.docx]

***S1 Table: Prevalence of morbidity amongst respondents of MAMMI and SIM surveys if they did report experiencing the condition in the 12 months prior to the pregnancy of their first child***

| **Urinary Incontinence** | **MAMMI – During** | **MAMMI - 3mths** | **MAMMI - 6mths** | **MAMMI - 9mths** | **MAMMI - 12mths** | **SIM - 6mths** | **SIM - 12mths** |
| --- | --- | --- | --- | --- | --- | --- | --- |
| Yes, did experience the condition | 162 (66.39%) | 124  (65.61%) | 102 (58.62%) | 104 (63.41%) | 82 (58.57%) | 6  (60%) | 2  (66.67%) |
| No, did not experience the condition | 82  (33.61%) | 65  (34.39%) | 72 (41.38%) | 60  (36.59%) | 58 (41.43%) | 4  (40%) | 1  (33.33%) |
| Total (n) | 244 | 189 | 174 | 164 | 140 | 10 | 3 |
|  |  |  |  |  |  |  |  |
| **Faecal**  **Incontinence** | **MAMMI – During** | **MAMMI - 3mths** | **MAMMI - 6mths** | **MAMMI - 9mths** | **MAMMI - 12mths** | **SIM - 6mths** | **SIM - 12mths** |
| Yes, did experience the condition | 5  (27.78%) | 2  (14.29%) | 2  (15.38%) | 3  (23.08%) | 0  (0%) | 0  (0%) | 0  (0%) |
| No, did not experience the condition | 13  (72.22%) | 12  (85.71%) | 11  (84.62%) | 10  (76.92% | 10 (100%) | 0  (0%) | 0  (0%) |
| Total (n) | 18 | 14 | 13 | 13 | 10 | 0 | 0 |
|  |  |  |  |  |  |  |  |
| **Pelvic Girdle Pain** | **MAMMI – During** | **MAMMI - 3mths** | **MAMMI - 6mths** | **MAMMI - 9mths** | **MAMMI - 12mths** | **SIM - 6mths** | **SIM - 12mths** |
| Yes, did experience the condition | 1,031 (89.34%) | 743  (82.74%) | 687 (81.59%) | 617 (81.83%) | 553 (81.56%) | 57 (87.69%) | 35 (83.33%) |
| No, did not experience the condition | 123  (10.66%) | 155  (17.26%) | 155 (18.41%) | 137 (18.17%) | 125 (18.44%) | 8  (12.31%) | 7  (16.67%) |
| Total (n) | 1,154 | 898 | 842 | 754 | 678 | 65 | 42 |

***S1 Table contd:* *Prevalence of morbidity amongst respondents of MAMMI and SIM surveys if they did report experiencing the condition in the 12 months prior to the pregnancy of their first child***

| **Sexual Health Problems** | **MAMMI – During** | **MAMMI - 3mths** | **MAMMI - 6mths** | **MAMMI - 9mths** | **MAMMI - 12mths** | **SIM - 6mths** | **SIM - 12mths** |
| --- | --- | --- | --- | --- | --- | --- | --- |
| Yes, did experience the condition | 1,181 (76.64%) | 984  (83.74%) | 974 (81.99%) | 851 (75.64%) | 749 (71.13%) | 74 (89.16%) | 46 (90.20%) |
| No, did not experience the condition | 360  (23.36%) | 191  (16.26%) | 214 (18.01%) | 274 (24.36%) | 304 (28.87%) | 9  (10.84%) | 5  (9.80%) |
| Total (n) | 1,541 | 1,175 | 1,188 | 1,125 | 1,053 | 83 | 51 |
|  |  |  |  |  |  |  |  |
| **Depression** | **MAMMI – During** | **MAMMI - 3mths** | **MAMMI - 6mths** | **MAMMI - 9mths** | **MAMMI - 12mths** | **SIM - 6mths** | **SIM - 12mths** |
| Yes, did experience the condition | 109 (35.97%) | 71 (29.83%) | 76 (34.55%) | 62 (31.63%) | 55 (30.05%) | 5  (45.45%) | 1  (14.29%) |
| No, did not experience the condition | 194 (64.03%) | 167 (70.17%) | 144 (65.45%) | 134 (68.37%) | 128 (69.95%) | 6  (54.55%) | 6  (85.71%) |
| Total (n) | 303 | 238 | 220 | 196 | 183 | 11 | 7 |
|  |  |  |  |  |  |  |  |
| **Anxiety** | **MAMMI – During** | **MAMMI - 3mths** | **MAMMI - 6mths** | **MAMMI - 9mths** | **MAMMI - 12mths** | **SIM - 6mths** | **SIM - 12mths** |
| Yes, did experience the condition | 85  (42.29%) | 52 (33.12%) | 41 (28.87%) | 33 (26.40%) | 26 (23.01%) | 2  (40%) | 1  (50%) |
| No, did not experience the condition | 116 (57.71%) | 105 (66.88%) | 101 (71.13%) | 92 (73.60%) | 87 (76.99%) | 3  (60%) | 1  (50%) |
| Total (n) | 201 | 157 | 142 | 125 | 113 | 5 | 2 |
