# Supplementary material for: The prevalence and persistence of maternal morbidities after first vs. second birth: A prospective cohort study in Ireland
Source: PLoS One. 2025 Oct 22;20(10):e0332891. doi: 10.1371/journal.pone.0332891 (PMC12543160; doi:10.1371/journal.pone.0332891)
Supplement: S2 Table — (DOCX) [file pone.0332891.s002.docx]

***S2 Table:* *Prevalence of morbidity amongst respondents of MAMMI and SIM surveys if they did not experience the condition in the 12 months prior to the pregnancy of their first child.***

| **Urinary Incontinence** | **MAMMI – During** | **MAMMI - 3mths** | **MAMMI - 6mths** | **MAMMI - 9mths** | **MAMMI - 12mths** | **SIM - 6mths** | **SIM - 12mths** |
| --- | --- | --- | --- | --- | --- | --- | --- |
| Yes, did experience the condition | 336  (12.21%) | 635 (28.59%) | 428 (20.62%) | 368 (19.03%) | 358 (20.16%) | 40 (25.97%) | 25 (23.15%) |
| No, did not experience the condition | 2,415 (87.79%) | 1,586 (71.41%) | 1,648 (79.38%) | 1,566 (80.97%) | 1,418 (79.84%) | 114 (74.03%) | 83 (76.85%) |
| Total (n) | 2,751 | 2,221 | 2,076 | 1,934 | 1,776 | 154 | 108 |
|  |  |  |  |  |  |  |  |
| **Faecal**  **Incontinence** | **MAMMI – During** | **MAMMI - 3mths** | **MAMMI - 6mths** | **MAMMI - 9mths** | **MAMMI - 12mths** | **SIM - 6mths** | **SIM - 12mths** |
| Yes, did experience the condition | 11  (0.37%) | 40  (1.66%) | 16  (0.71%) | 18  (0.86%) | 14  (0.72%) | 4  (2.40%) | - |
| No, did not experience the condition | 2,979 (99.63%) | 2,374 (98.34%) | 2,239 (99.29%) | 2,083 (99.14%) | 1,933 (99.28%) | 163 (97.60%) | 113  (100%) |
| Total (n) | 2,990 | 2,414 | 2,255 | 2,101 | 1,947 | 167 | 113 |
|  |  |  |  |  |  |  |  |
| **Pelvic Girdle Pain** | **MAMMI – During** | **MAMMI - 3mths** | **MAMMI - 6mths** | **MAMMI - 9mths** | **MAMMI - 12mths** | **SIM - 6mths** | **SIM - 12mths** |
| Yes, did experience the condition | 402 (48.67%) | 427 (66.61%) | 383 (61.38%) | 334 (62.20%) | 286 (58.97%) | 34 (70.83%) | 19 (70.37%) |
| No, did not experience the condition | 424 (51.33%) | 214 (33.39%) | 241 (38.62%) | 203 (37.80%) | 199 (41.03%) | 14 (29.17%) | 8  (29.63%) |
| Total (n) | 826 | 641 | 624 | 537 | 485 | 48 | 27 |

***S2 Table contd:* *Prevalence of morbidity amongst respondents of MAMMI and SIM surveys if they did not experience the condition in the 12 months prior to the pregnancy of their first child.***

|  |  |  |  |  |  |  |  |
| --- | --- | --- | --- | --- | --- | --- | --- |
| **Sexual Health Problems** | **MAMMI – During** | **MAMMI - 3mths** | **MAMMI - 6mths** | **MAMMI - 9mths** | **MAMMI - 12mths** | **SIM - 6mths** | **SIM - 12mths** |
| Yes, did experience the condition | 683 (50.59%) | 684 (71.62%) | 577 (61.45%) | 453 (51.59%) | 378 (46.32%) | 40 (65.57%) | 31 (63.27%) |
| No, did not experience the condition | 667 (49.41%) | 271 (28.38%) | 362 (38.55%) | 425 (48.41%) | 438 (53.68%) | 21 (34.43%) | 18 (36.73%) |
| Total (n) | 1,350 | 955 | 939 | 878 | 816 | 61 | 49 |
|  |  |  |  |  |  |  |  |
| **Depression** | **MAMMI – During** | **MAMMI - 3mths** | **MAMMI - 6mths** | **MAMMI - 9mths** | **MAMMI - 12mths** | **SIM - 6mths** | **SIM - 12mths** |
| Yes, did experience the condition | 294 (7.25%) | 214 (9.74%) | 203 (9.91%) | 184 (9.60%) | 173 (9.70%) | 25 (16.13%) | 14 (13.59%) |
| No, did not experience the condition | 2,483 (92.75%) | 1,983 (90.26%) | 1,846 (90.09%) | 1,733 (90.40%) | 1,611 (90.30%) | 130 (83.87%) | 89 (86.41%) |
| Total (n) | 2,777 | 2,197 | 2,049 | 1,917 | 1,784 | 155 | 103 |
|  |  |  |  |  |  |  |  |
| **Anxiety** | **MAMMI – During** | **MAMMI - 3mths** | **MAMMI - 6mths** | **MAMMI - 9mths** | **MAMMI - 12mths** | **SIM - 6mths** | **SIM - 12mths** |
| Yes, did experience the condition | 369 (13.26%) | 173 (7.60%) | 175 (8.25%) | 127 (6.38%) | 159 (8.55%) | 18 (11.18%) | 13 (12.04%) |
| No, did not experience the condition | 2,413 (86.74%) | 2,103 (92.40%) | 1945 (91.75%) | 1,864 (93.62%) | 1,701 (91.45%) | 143 (88.82%) | 95 (87.96%) |
| Total (n) | 2,782 | 2,276 | 2,120 | 1,991 | 1,860 | 161 | 108 |
